# Secondary compounds of *Pinus massoniana* alter decomposers' effects on *Quercus variabilis* litter decomposition

**DOI:** 10.1002/ece3.4433

**Published:** 2018-08-29

**Authors:** Hong Lin, Yunxia Zhao, Numaimaiti Muyidong, Kai Tian, Zaihua He, Xiangshi Kong, Shucun Sun, Xingjun Tian

**Affiliations:** ^1^ School of Life Sciences Nanjing University Nanjing China; ^2^ Co‐Innovation Center for Sustainable Forestry in Southern China Nanjing Forestry University Nanjing China

**Keywords:** aqueous extracts, isopod, litter decomposition, plant–soil interactions, secondary compounds, tannins

## Abstract

A major gap to understand the effects of plant secondary compounds on litter decomposition in the brown food web is lack of information about how these secondary compounds modify the activities of soil decomposers. To address this question, we conducted an experiment where aqueous extracts and tannins prepared from *Pinus massoniana* needles were added to soils collected either from *P. massoniana* (pine soil) or *Quercus variabilis* (oak soil). Our objective was to investigate the cascading effects of the two compounds on isopod (*Armadillidium vulgare*) activity and subsequent change in *Q. variabilis* litter decomposition. We found that in pine soil, both aqueous extracts and tannins (especially at high concentrations) had positive effects on litter decomposition rates when isopods were present. While without isopods, litter decomposition was enhanced only by high concentrations of aqueous extracts, and tannins had no significant effect on decomposition. In oak soil, high concentrations of aqueous extracts and tannins inhibited litter decomposition and soil microbial biomass, regardless of whether isopods were present or not. Low concentrations of aqueous extracts increased litter decomposition rates and soil microbial biomass in oak soil in the absence of isopods. Based on our results, we suggest that the high concentration of secondary compounds in *P. massoniana* is a key factor influencing the effects of decomposers on litter decomposition rates, and tannins form a major part of secondary compounds. These funding particularly provide insight into form‐ and concentration‐oriented effects of secondary compounds and promote our understanding of litter decomposition and soil nutrient cycling in forest ecosystem.

## 1 INTRODUCTION

Leaf secondary compounds are widely recognized as a key driver of plant litter decomposability (Cornwell et al., 2008) playing a major role in litter decomposition and nutrient cycling (Chomel et al., 2016). They may influence litter decomposition directly through toxic effects limiting the growth and activity of decomposers. For example, several studies have shown secondary compounds to have important inhibitive effects on fungal colonization, soil microorganism respiration, and enzymatic activity (Chomel et al., 2014; White, 1986, 1991, 1994). Secondary compounds can also affect litter decomposition indirectly. For example, phenolic compounds can decrease the palatability of leaf litter for soil micro‐arthropods (Asplund, Bokhorst, & Wardle, 2013; Levin, 1976), causing adverse effects on litter decomposition. In addition, secondary compounds can form recalcitrant complexes with proteins, which inhibit soil enzyme activity and impede the decomposition of organic matter (Cadisch & Giller, 1997; Chomel et al., 2016; Kraus, Dahlgren, & Zasoski, 2003; Madritch & Lindroth, 2015; Ushio, Balser, & Kitayama, 2012). Tannins, water‐soluble polyphenolic compounds, are rich in woody plants, especially in pine (Hättenschwiler & Vitousek, 2000; Kraus et al., 2003). Like many other secondary compounds, tannins have been shown having a major role in litter decomposition and nutrient availability (Cornelissen, Stiling, & Drake, 2004; Hättenschwiler & Vitousek, 2000). However, it still remains difficult to study the effects of secondary compounds on decomposition process owing to the very broad diversity of secondary compounds. Also, few studies have addressed the interactive effects of secondary compounds and soil fauna on litter decomposition (Das & Joy, 2009; Hwang & Lindroth, 1997; Whitham et al., 2006).

Isopods (*Armadillidium vulgare*, order: *Isopoda*, family*: Oniscidea*) are saprophagous invertebrates that are dominant members of soil fauna communities (David & Handa, 2010; Zimmer, 2002). Isopods may have average densities as high as 10,000 individuals/m^2^ in the USA (Frouz et al., 20042004) and 100–500 individuals/m^2^ in Nanjing, China (Hong, Boping, & Tian, 1994). They are voracious detritivores that mechanically break apart plant litter and increase the contact surface area with soil during decomposition (Seastedt, 1984). Thus, their feeding activities can accelerate litter decomposition (David & Handa, 2010; Jia et al., 2015). In addition, through alterations to the soil microenvironment (caused by their feeding, migration, etc.), the soil fauna may also influence the abundance of extracellular enzymes, microbial activity, and microbial biomass in the soil; microbial growth may be stimulated by the frass produced by soil fauna (Chomel, Guittonny‐Larchevêque, DesRochers, & Baldy, 2015; David, 2014; Jia et al., 2015). However, how secondary compounds affect the isopods activity in litter decomposition and the roles of isopods and microorganism are still not clear.

Thus, the objective of this study was to determine how leaf secondary compounds alter the relative importance of decomposers associated with litter decomposition, and so regulate soil nutrient cycling. We test two hypotheses: (a) the addition of aqueous extracts and tannins will inhibit the effect of decomposers, including isopods and microorganisms on *Quercus variabilis* (order: *Fagales*, family: *Fagacea*) litter decomposition, via known allelopathic and toxic effects (Chomel et al., 2014; Hättenschwiler & Vitousek, 2000); and (b) addition treatments will vary depending on soil source (whether oak or pine), being more significant in oak soil due to its higher quality for soil organisms activity (Ushio et al., 2012). To test these hypotheses, we established a laboratory experiment to compare the effects of aqueous extracts and one main family of secondary compounds, tannins from *Pinus massoniana* (order: *Coniferales*, family: *Pinaceae*) on decomposition rates of *Q. variabilis* litter (these two species dominate the mixed conifer‐broadleaf forests of the study area in Nanjing, China).

## 2 MATERIALS and METHODS

### 2.1 Study site

We collected soils from a mixed conifer‐broadleaf forest (two dominant tree species: *P. massoniana* and *Q. variabilis*) on Zijin Mountain (447.1 m asl, 32°5′N, 118°48′E) which is located in Nanjing, Jiangsu, China. This area has a subtropical monsoon climate with a mean annual precipitation of 1,106.5 mm (distributed from June to July), and a mean annual air temperature of 15.4°C (min: 1.9°C in January; max: 28.2°C in July). Soils are classified as humic cambisols that are slightly acidic with a pH of 5.0 ± 0.02 (FAO‐UNESCO, 1987). The bedrock is formed of sandstone and shale, and the soil humus layer is rich in nutrients and organic matter.

### 2.2 Decomposition experiment design

Litter samples collected from *Q. variabilis* were allowed to decompose in a laboratory microcosm. Air‐dried *Q. variabilis* litter (0.5 ± 0.02 g) was mixed with 40 g field‐collected soil, from either oak or pine tree stands, and placed in plastic incubation boxes with a basal area of 75 cm^2^ each; boxes were covered with ventilated lids. All incubation boxes were divided into two groups: one group with isopods and the other without isopods. In the group with isopods, two isopods were placed in each box to simulate average isopod density (Zijin mountain, approximately 180 individuals/m^2^) (Jia et al., 2015). Incubation boxes were checked every week, and dead isopods were replaced by similar sized ones from the container with a corresponding food source and were then tagged in order to count the total number of dead isopods. The group without isopods contained only *Q. variabilis* litter with either pine‐ or oak‐derived soil. In both groups (with or without isopods) and for both soil types, either aqueous extracts prepared from *P. massoniana* litter or extracted tannins were added every month, at one of two concentrations (high or low, as described in 2.4). A total of 5 ml of extract were added each time, to mimic *P. massoniana* litter production in the study plots, which averaged 70 g m^−2^ month^−1^. Control boxes were treated with distilled water only. Thus, there were five treatments in total: distilled water (control), aqueous extracts at high (high aqueous) or low (low aqueous) concentration, and tannins at high (high tannins) or low (low tannins) concentration. Overall, the experiment comprised 480 incubation boxes (2 groups × 2 soil types × 5 treatments × 4 replicates × 6 collection times). All incubation boxes were kept at 25°C and soils maintained at a gravimetric moisture content of 50%–60% during the experiment.

Incubation boxes were harvested for analysis every month from April to September 2015. At each timepoint, 80 boxes were harvested, with litter and soils placed into separate polyethylene bags. To determine *Q. variabilis* litter mass loss, any remaining soil was carefully separated from the litter, then the litter samples were oven‐dried at 60°C to a constant weight (about 1 week). Soil samples were stored at a constant moisture level before measuring the pH, carbon and nitrogen contents, soil microbial respiration rate, and enzymatic activity.

### 2.3 Collection of soil and leaf litter samples

In October and November of 2014, freshly senescent leaves of *P. massoniana* and *Q. variabilis* were collected from four independent plots (2 m × 2 m) with approximately 10 m spacing between adjacent plots, and air‐dried for 1 month until samples achieved a constant weight. The mineral layer (0–5 cm) of the soil was sampled in the four plots below *P. massoniana* and *Q. variabilis* individuals, and samples sieved through 2‐mm mesh. Prior to use, soil samples were maintained at a stable 20°C in the dark with constant humidity.

### 2.4 Preparation of aqueous extracts from *P. massoniana* litter and tannin extract

Air‐dried *P. massoniana* leaf litter was cut into 0.2–0.5 cm pieces then soaked in distilled water (1 g per 10 ml) for 48 hr to prepare aqueous extracts. The solution was filtered and further diluted in distilled water to two concentrations (g/ml): 0.002 (low) and 0.100 (high) (Zhang, Zhang, Zou, & Siemann, 2014). Solutions were kept in a refrigerator at 4°C until needed.

Tannins were extracted using an ultrasonic assisted technique as described by Yang Jing, Ning, Min, and Jian (2013). Leaves collected from *P. massoniana* were freeze‐dried (yielding 100 g of dried leaves total), finely ground, and extracted three times with 70% acetone (liquid–solid ratio: 20:1 [m:g]) using ultrasound equipment, with each extraction lasting 50 min (Power: 300W, temperature: 30°C). The three 70% acetone fractions were combined and concentrated by evaporation with a rotary evaporator. The extracted tannins were measured using a spectrophotometer (Saxena, Mishra, Vishwakarma, & Saxena, 2013) and had a total concentration of 95.4%. The prepared tannins were diluted with distilled water to two concentrations (g/ml): 0.0001 (low) and 0.006 (high). Extracts were kept in a refrigerator at 4°C until incubation.

### 2.5 Collection of isopods

Adult isopods (body length: 8–10 mm) were hand‐collected in March 2015 from study plots. Individuals were taken back to the laboratory and cultured in a 5‐L plastic box. Isopods were fed *Q. variabilis* litter, and kept in the dark at 20°C with appropriate soil moisture. After 2 weeks, lively individuals were selected for the decomposition experiment.

### 2.6 Measurements of soil chemical and microbial properties

Prior to the experiment, the chemical properties of leaf litter and soil samples were determined using 2 g of material oven‐dried at 60°C for 48 hr. The total C and N concentrations in both dried litter and soil samples were determined using an elemental analyzer (Elemental Vario MICRO, Germany). The lignin concentration of the litter samples was determined by gravimetric analysis of a hot sulfuric acid digestion (Osono & Takeda, 2002). A glass electrode was used to measure the pH of soil samples in water (1:2.5 soil to water ratio), after shaking the solution for approximately 30 min (Dick, Cheng, & Wang, 2000) (Supporting Information Table S1).

Soil microbial biomass and enzymatic activities were measured to monitor the functional responses of microorganisms to isopods. Soil microbial biomass was measured using the substrate‐induced respiration (SIR) method (Osono & Takeda, 2002). All soil samples were maintained at 60% dry weight to avoid water limitation. Subsamples of 1 g of fresh soil were then placed into 100 ml glass vials. Next, 1 ml of an aqueous glucose solution (10 mg glucose per 1 g of soil) was added to each vial. The vials were then sealed and incubated at 25°C for 1 hr. Finally, carbon dioxide production (by soil microbes) was assayed using an infrared gas analyzer (Bailey et al., 20092002).

Extracellular enzymes responsible for carbon cycling (cellobiohydrolase, CBH1; b‐1,4‐glucosidase, BG; and b‐1,4‐xylosidase, BX), nitrogen cycling (nitrate reductase, NR; urease, URE), phosphorus cycling (acid phosphatase, ACP; alkaline phosphatase, ALP), and polyphenol metabolism (phenol oxidase, PhOx; peroxidase, Pero) were quantified spectrophotometrically.

The activity of the enzymes CBH1 (E.C. 3.2.1.91), BG (E.C. 3.2.1.21) and BX (E.C. 3.2.1.37) was determined using 1.2 mM 4‐nitrophenyl‐b‐D‐linked (PNPX) substrates (cellobioside, glucopyranoside, and xylopyranoside), with soil samples and substrates incubated together in the dark at 40°C for 1.5 hr (pH 5.0; 0.2 M Na_2_CO_3_ was added to stop the reaction). Concentrations of 4‐Nitrophenyl (PNP) were quantified by measuring absorbance at 400 nm using a microplate spectrophotometer (Tecan Safire2, Switzerland), with samples placed in 96‐well plates (Vepsalainen, Kukkonen, Vestberg, Sirvio, & Niemi, 2001). All measures of enzymatic activity are expressed in μmol PNP hr^−1^ g^−1^ soil.

The activity of PhOx (E.C. 1.10.3.2) and Pero (E.C. 1.11.1.7) enzymes was measured spectrophotometrically using 50 μl of 25 mM 1‐3,4‐dihydroxyphenylalanine (l‐DOPA) as the substrate, with incubation at 28°C for 1 hr (pH 5.5). Pero assays had 10 μl of 0.3% H_2_O_2_ added before measurement. Enzymatic activity was quantified by measuring absorbance at 450 nm using a microplate spectrophotometer and 96‐well plates (Saiyacork, Sinsabaugh, & Zak, 2002). Enzymatic activity is expressed in μmol l‐DOPA hr^−1^ g^−1^ soil.

Soil NR (E.C. 1.7.99.4) activity was determined using 200 mM KNO_3_ solution as a substrate, with incubation at room temperature for 30 min (pH 7.5; NO_2_^−^). Concentrations were determined with a spectrophotometer (Jing Hua, Shanghai, China) at a wavelength of 520 nm. Enzymatic activity was quantified by reference to a calibration curve; the curve was obtained from a soil incubation experiment carried out under identical conditions to those described above. Enzymatic activity is expressed in μg NO_2_^−^ min^−1^ g^−1^ soil (Daniel & Curran, 1981).

Soil URE activity was determined using urea as substrate, with samples incubated at 37°C and a pH of 6.7 (in 0.2 M phosphate buffer) for 24 hr. Absorbance was measured at 578 nm with a spectrophotometer (Nannipieri, Ceccanti, Cervelli, & Matarese, 1980), and enzymatic activity is expressed in mg NH_3_‐N hr^−1^ g^−1^ soil.

Finally, the enzymatic activity of ACP (E.C. 3.1.3.2) and ALP (E.C. 3.1.3.1) was determined in a 0.5% disodium phenyl phosphate solution incubated at 37°C for 24 hr (pH 5.0 for acid phosphatase; pH 10.0 for alkaline phosphatase; phenol concentration was determined with a spectrophotometer at 570 nm). Again, enzymatic activity was quantified by reference to a calibration curve obtained from a previous trial and is expressed in mg P hr^−1^ g^−1^ soil (Kandeler, Tscherko, & Spiegel, 1999).

### 2.7 Data analyses

The proportion of the substrate remaining over time was fit to a negative exponential model (*y* = e^*−kt*^) following Olson (1963), where *y* is the proportion of initial mass remaining at time *t*, and *k* is the litter decomposition rate constant (month^−1^). The best fit model was determined using Akaike's information criteria (AIC_c_), where a difference between two candidate models of ≥3 was used to indicate a significant difference in model fit (Hobbie et al., 2012).

Data were checked for deviations from normality and homogeneity of variance before analysis by Shapiro–Wilk test and quantile–quantile Plot. Data were log‐transformed to improve normality; for example, transformation was necessary for data on isopod deaths and extracellular enzyme activity. An analysis of variance (ANOVA) and Tukey's HSD (honest significant difference) test were applied to assess differences among treatments. Three‐ways ANOVAs were used to determine the effects of soil type, isopods activity, and the extract treatments on *Q. variabilis* litter decomposition. Extracellular enzyme activity was analyzed using repeated‐measures ANOVA, with treatment as the main effect and sampling time as a repeated factor. Repeated‐measures ANOVA was also used to compare treatment effects on litter mass loss and soil microbial biomass (SIR) over time. All statistical analyses were performed in SPSS (Version 19.0).

## 3 RESULTS

### 3.1 Litter decomposition rate

**Figure 1:**
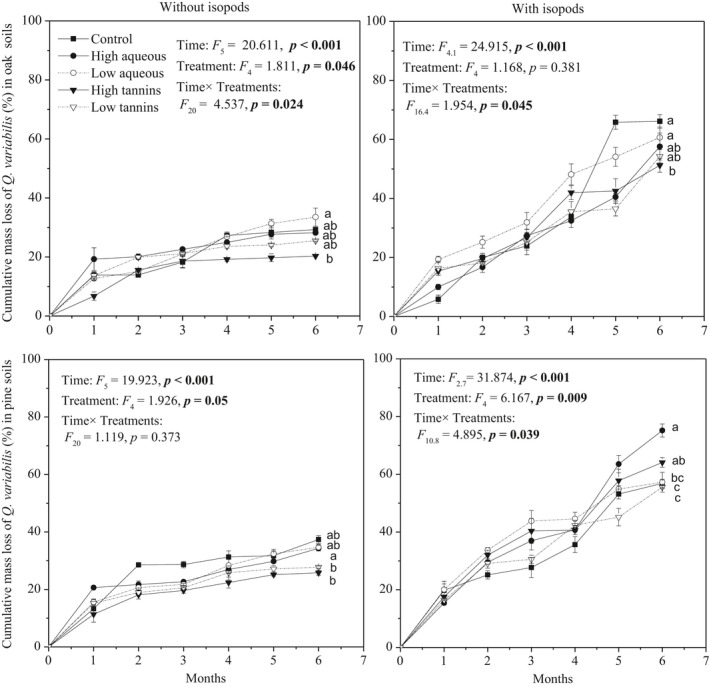
Effects of treatments (control, high aqueous, low aqueous, high tannins, and low tannins) on cumulative mass loss of *Quercus variabilis* litter with or without isopods (*Armadillidium vulgare*) in two soil types (oak soil and pine soil). Data with different letters indicate a significant difference (*p* < 0.05) from repeated‐measure ANOVA. Error bars indicate standard deviation (*SD*,* n* = 4)

**Figure 2:**
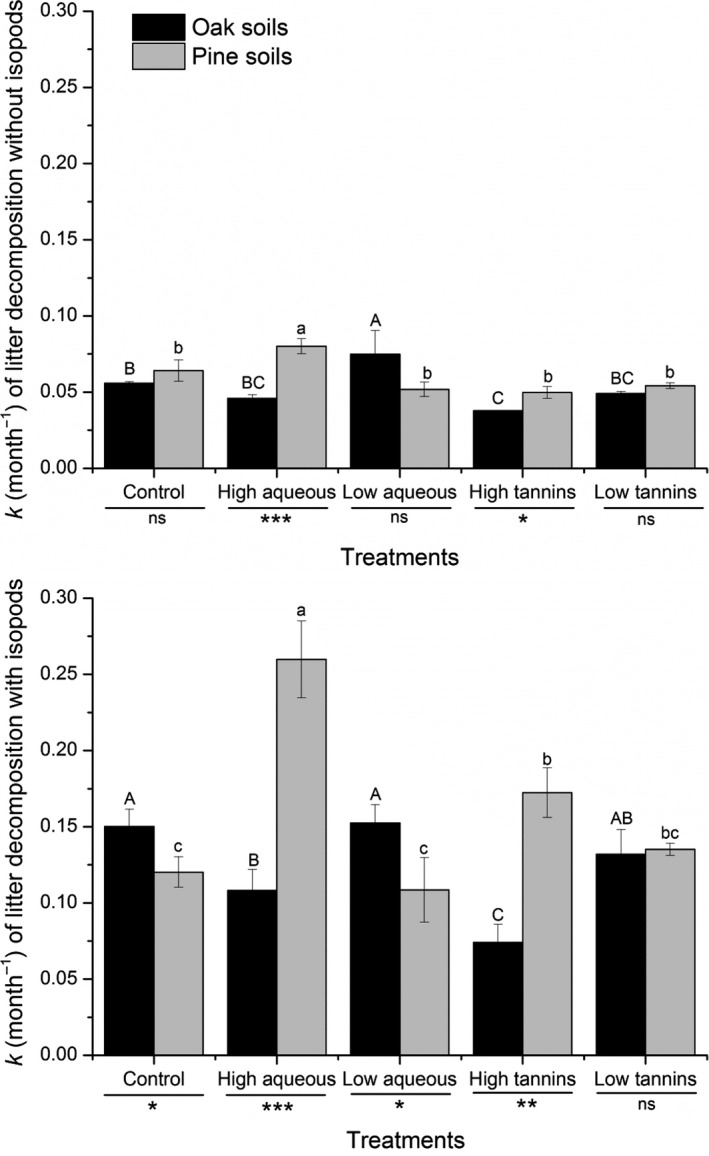
Decomposition rate (mean *k* values, month^−1^) of *Quercus variabilis* litter with or without isopods (*Armadillidium vulgare*) in two soil types (oak soil and pine soil) under different treatments (control, high aqueous, low aqueous, high tannins, and low tannins). Data with different letters indicates a significant difference (*p* < 0.05). *p < 0.05, **p < 0.01 and ***p < 0.001 by *t* test. Error bars indicate standard deviation (*SD*,* n* = 4)

Over the course of the decomposition experiment, in both oak‐ and pine‐derived soils, the cumulative mass of *Q. variabilis* litter lost (to decomposition) increased over the first 4 months, but the rate of loss then slowed in the final 2 months (Figure 1). Litter decomposed more slowly in the absence of isopods in all cases (*p *<* *0.001, Figures 1 and 2). The decomposition rate (*k* value) differed significantly between boxes with and without isopods (*p *<* *0.001, *F*_1_ = 136.95) and among extract treatments (*p *=* *0.036, *F*_4_ = 2.86) (Table 1).

**Table 1:** Three‐ways ANOVAs on the effects of soil types (oak soil or pine soil), fauna activity (with or without isopods, *Armadillidium vulgare*), treatments (control, high aqueous, low aqueous, high tannins and low tannins), and their interactions on the decomposition constant *k* during *Quercus variabilis* litter decomposition

	*df*	*F*	*p*
Soil types	1	26.35	**<0.001**
Fauna activity	1	136.95	**<0.001**
Treatment	4	2.86	**0.036**
Soil type × Fauna activity	1	2.94	0.094
Soil type × Treatment	4	3.676	**0.012**
Fauna activity × Treatment	4	1.891	0.131
Soil type × Fauna activity × Treatment	4	4.125	**0.007**

*Note*

*p* Values equal to or lower than 0.05 are in boldface.

Overall, in both soil types, the *Q. variabilis* litter decomposition rate was higher with rather than without isopods (Figure 2). In oak soil without isopods, the addition of a low concentration of aqueous extracts significantly increased the decomposition rate, while addition of a high concentration of tannins decreased the decomposition rate (Figure 2). Meanwhile, the *Q. variabilis* litter decomposition rate was enhanced by addition of a high concentration of aqueous extracts in pine soil without isopods (Figure 2). Soil types differed in how decomposition rate responded to the high aqueous and high tannin extract treatments (Figure 2). In oak soil with isopods, the decomposition rate decreased significantly in these treatments compared to controls, by 27.9% (*p *<* *0.05; high aqueous) and 50.6% (*p *<* *0.01; high tannins), respectively. Meanwhile in pine soil with isopods, the high aqueous treatment significantly increased (by 1.16‐fold) the decomposition rate compared to controls (Figure 2). With the exception of the low concentration tannin treatment, most treatments differed in their effects on litter decomposition rate between soil types (Figure 2). However, there was no interaction between treatment and the presence/absence of isopods. The three‐way interaction between treatment, isopod presence/absence, and soil type was significant though (*p *=* *0.007, *F*_4_ = 4.125) (Table 1).

### 3.2 Effects of secondary compounds on soil C, N, and pH

After the 6‐month incubation period, in oak soil without isopods, measures of C and N content were highest in the low aqueous extract treatment and lowest in the low tannin treatment; meanwhile, with isopods, the addition of either aqueous extracts or tannins reduced C and N content compared to controls (*p *<* *0.05, Table 2). In pine soil without isopods, most treatments increased soil C and N content, with the highest C content observed in the high aqueous extract treatment (Table 2). Meanwhile, with isopods, the opposite pattern was observed, with most treatments decreasing soil C and N content; C and N were lowest in the high aqueous extract treatment (*p *<* *0.05, Table 2).

**Table 2:** C and N content with aqueous extracts and tannins addition in the two given soils (oak soil or pine soil), with or without isopods (*Armadillidium vulgare*) after the 6 months litter decomposition

	Control	High aqueous	Low aqueous	High tannins	Low tannins
Oak soil					
Without isopods					
C (%)	4.96 ± 0.02^b^	4.9 ± 0.02^b^	5.33 ± 0.03^a^	5.11 ± 0.01^ab^	4.05 ± 0.02^c^
N (%)	0.33 ± 0.003^a^	0.31 ± 0.002^a^	0.34 ± 0.005^a^	0.33 ± 0.002^a^	0.27 ± 0.002^b^
With isopods					
C (%)	7.25 ± 0.03^a^	5.05 ± 0.02^bc^	4.74 ± 0.01^c^	5.57 ± 0.05^b^	4.89 ± 0.02^c^
N (%)	0.43 ± 0.004^a^	0.33 ± 0.002^b^	0.30 ± 0.002^b^	0.33 ± 0.001^b^	0.33 ± 0.03^b^
Pine soil					
Without isopods					
C (%)	5.27 ± 0.01^c^	6.57 ± 0.02^a^	5.76 ± 0.01^b^	6.16 ± 0.05^ab^	5.78 ± 0.02^b^
N (%)	0.30 ± 0.005^a^	0.38 ± 0.005^a^	0.34 ± 0.003^a^	0.34 ± 0.005^a^	0.33 ± 0.002^a^
With isopods					
C (%)	7.22 ± 0.05^a^	5.29 ± 0.02^c^	5.97 ± 0.01^b^	6.94 ± 0.02^ab^	5.46 ± 0.01^c^
N (%)	0.41 ± 0.005^a^	0.33 ± 0.003^b^	0.37 ± 0.002^b^	0.40 ± 0.001^a^	0.34 ± 0.002^b^

*Note*

Data with different superscript letters in row are significantly different (*p *<* *0.05, *n *=* *4).

The soil pH generally decreased over time and there was a treatment effect. In both soil types, the pH was higher in all treatment boxes compared to controls, with the exception of the high tannin treatment; there was no effect of isopod presence. The highest mean pH occurred in the low concentration aqueous extract treatment (*p *<* *0.01; Supporting Information Tables S1 and S2) for both soil types and regardless of isopod presence or absence.

### 3.3 Effects of secondary compounds on soil microbial biomass and isopods

**Figure 3:**
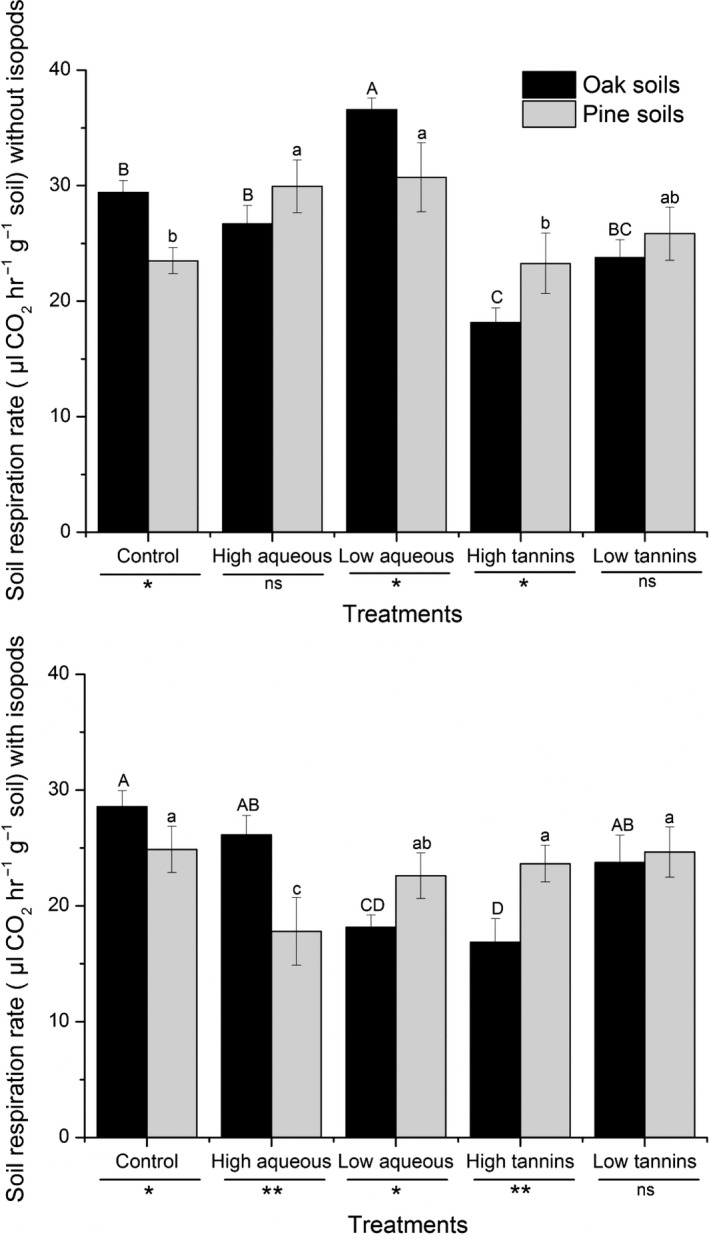
The mean of substrate‐induced respiration (SIR rate, μl CO_2_ hr^−1^ g^−1^ soil) with or without isopods (*Armadillidium vulgare*) in two soil types (oak soil and pine soil) under different treatments (control, high aqueous, low aqueous, high tannins, and low tannins). Data with different letters indicates a significant difference (*p* < 0.05) from repeated‐measure ANOVA. *p < 0.05, **p < 0.01 and ***p < 0.001 by *t* test. Error bars indicate standard deviation (*SD*,* n* = 4)

Substrate‐induced respiration was used to estimate the soil microbial biomass. In the case without isopods, in oak soil, the addition of a low concentration of aqueous extracts significantly increased SIR by 24.3% compared to controls, while high tannin concentrations decreased SIR by 38.3% (Figure 3). In pine soil, SIR also increased compared to controls with the addition of aqueous extracts, by 27.5% in the high aqueous treatment (*p *=* *0.008) and 30.8% in the low aqueous treatment (*p *=* *0.003); tannin addition had no effect on SIR. In the case with isopods, in oak soil, the addition of a low concentration of aqueous extracts decreased SIR by 36.5% (*p *<* *0.01), and a high concentration of tannins also significantly inhibited SIR (40% decrease; *p *<* *0.001). In pine soil, most treatments had no effect on SIR (Figure 3), with the exception of the high aqueous treatment which inhibited SIR.

**Figure 4:**
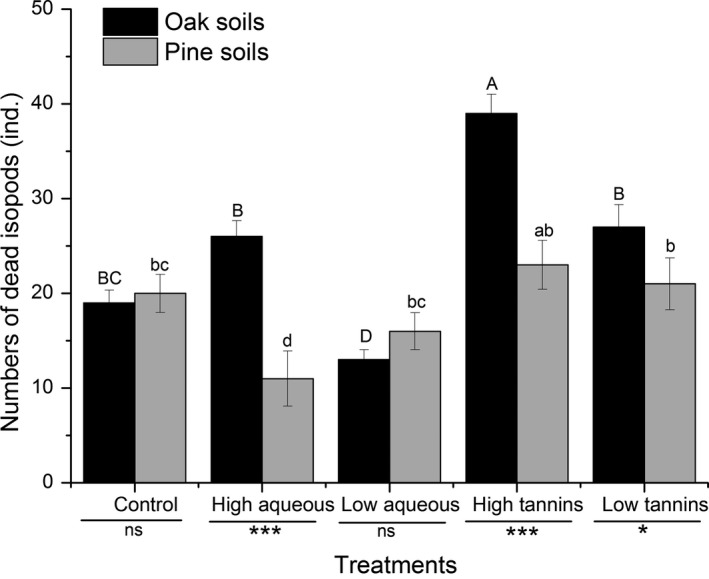
The total numbers of dead isopods (*Armadillidium vulgare*) (ind.) in two soil types (oak soil and pine soil) under different treatments (control, high aqueous, low aqueous, high tannins, and low tannins). Data with different letters indicates a significant difference (*p* < 0.05). *p < 0.05, **p < 0.01 and ***p < 0.001 by *t* test. Error bars indicate standard deviation (*SD*,* n* = 4)

The isopod death rate responded to treatments differently in the two soil types. In oak soil, fewer deaths occurred in the low aqueous treatment (compared to controls, see Figure 4), but more isopods died in the presence of high tannin concentrations. In pine soil, fewer deaths occurred with high aqueous extract concentrations (Tukey's test, *p *<* *0.05). Treatment effects (for high aqueous, high tannins, and low tannins) on isopod deaths differed significantly between soil types (Figure 4).

### 3.4 Effects of secondary compounds on soil extracellular enzymes

The interaction between soil type, isopod presence/absence, and extract treatment was significant for most soil extracellular enzymes (Tables 3 and 4). In oak soil without isopods, the addition of aqueous extracts inhibited the activity of most soil enzymes; one exception was that high concentrations of aqueous extracts promoted ALP and URE activity (Tukey's test, *p *<* *0.05). Meanwhile, treatment with tannins inhibited the activity of soil extracellular enzymes involved in N cycles (NR, URE) and C cycles (BG, BX) (Tukey's test, *p *<* *0.05), but promoted ALP activity. With isopods (and oak soil), high aqueous extract concentrations now enhanced rather than inhibited the activity of most soil enzymes, while low concentrations of extracts or tannins reduced enzyme activity. In pine soil without isopods, Perox and Phox activity was higher when high concentrations of aqueous extracts or tannins were added (Tukey's test, *p *<* *0.05), but those enzymes involved in N cycling (NR, URE) were inhibited. With isopods, aqueous extract and tannin treatments inhibited C cycle enzymes, and high concentrations of either also reduced the activity of Perox and Phox enzymes (Tukey's test, *p *<* *0.05).

**Table 3:** Effects (indicated by *p* values from repeated‐measures ANOVA) of sampling time, soil types (oak soil or pine soil), fauna activity (with or without isopods, *Armadillidium vulgare*), treatments (control, high aqueous, low aqueous, high tannins, and low tannins), and their interactions on soil enzyme activities during 6 months litter decomposition

Variation	NR	ALP	ACP	URE	BG	BX	CBH1	Pero	PhOx
Between subjects									
Intercept	**<0.001**	**<0.001**	**<0.001**	**<0.001**	**<0.001**	**<0.001**	**<0.001**	**<0.001**	**<0.001**
Soil type	**<0.001**	**<0.001**	**0.001**	**<0.001**	**0.029**	0.234	0.525	0.092	0.845
Treatment	**<0.001**	**<0.001**	**<0.001**	**0.003**	**0.021**	**0.02**	**0.011**	0.715	**0.049**
Fauna activity	**0.05**	**0.032**	0.2	**<0.001**	**0.03**	**0.049**	**<0.001**	**0.05**	0.154
Soil type × Treatment	**0.005**	0.303	**<0.001**	0.174	0.181	0.116	**<0.001**	**0.002**	0.696
Soil type × Fauna activity	**0.017**	0.391	0.204	0.683	0.423	0.579	0.357	0.699	0.608
Treatment × Fauna activity	**0.05**	0.694	0.573	**0.011**	0.85	0.791	0.638	0.567	0.671
Soil type × Treatment × Fauna activity	0.57	0.389	0.161	0.24	**0.016**	**<0.001**	0.888	0.439	0.315
Within subjects									
Time	**<0.001**	**<0.001**	**<0.001**	**<0.001**	**<0.001**	**<0.001**	**<0.001**	**<0.001**	**<0.001**
Time × Soil type	**<0.001**	**<0.001**	**<0.001**	**<0.001**	0.159	**<0.001**	0.311	**<0.001**	**<0.001**
Time × Treatment	**<0.001**	**<0.001**	**0.008**	**0.049**	**0.037**	**0.003**	0.426	**<0.001**	0.368
Time × Fauna activity	**0.003**	0.326	0.148	**0.046**	0.412	0.954	**<0.001**	**0.047**	**0.05**
Time × Soil type × Treatment	**0.006**	0.113	**<0.001**	**0.004**	0.317	**0.009**	**<0.001**	**<0.001**	**0.007**
Time × Soil type × Fauna activity	0.087	**0.011**	0.87	0.353	0.736	0.454	**0.044**	0.84	0.561
Time × Treatment × Fauna activity	0.108	**0.048**	0.491	**0.008**	0.632	0.815	0.831	**0.001**	0.992
Time × Soil type × Treatment × Fauna activity	**0.026**	**<0.001**	0.884	**0.019**	**0.023**	**0.015**	0.243	**0.015**	**0.038**

*Notes*

*p* Values equal to or lower than 0.05 are in boldface.

ACP: acid phosphatase; ALP: alkaline phosphatase; BG: b‐1,4‐glucosidase; BX: b‐1,4‐xylosidase; CBH1: cellobiohydrolase; NR: nitrate reductase; Pero: peroxidase; PhOx: phenol oxidase; URE: urease.

**Table 4:** Effects of fauna activity (with or without isopods, *Armadillidium vulgare*) and treatments (control, high aqueous, low aqueous, high tannins and low tannins) on soil extracellular enzyme activities in two given soils (oak soil or pine soil). Data represent mean values of 6 months sampling during litter decomposition and standard error (*n* = 4)

	Without isopods					With isopods				
	Control	High aqueous	Low aqueous	High tannins	Low tannins	Control	High aqueous	Low aqueous	High tannins	Low tannins
Oak soil										
NR (IU)	13.30 ± 0.42^a^	13.08 ± 0.58^a^	12.56 ± 0.17^ab^	10.87 ± 0.52^c^	11.19 ± 0.35^bc^	14.38 ± 0.12^ab^	15.15 ± 0.48^a^	12.82 ± 0.59^b^	12.43 ± 0.29^bc^	10.57 ± 0.54^c^
ALP (IU)	13.25 ± 0.21^b^	14.69 ± 0.35^a^	13.57 ± 0.01^b^	14.97 ± 0.02^a^	12.96 ± 0.19^b^	13.41 ± 0.50^abc^	14.02 ± 0.15^ab^	13.06 ± 0.20^bc^	14.27 ± 0.26^a^	12.78 ± 0.34^c^
ACP (IU)	11.19 ± 0.07^a^	11.13 ± 0.09^a^	10.98 ± 0.08^a^	11.03 ± 0.03^a^	10.17 ± 0.02^b^	11.06 ± 0.21^a^	11.01 ± 0.20^a^	10.11 ± 0.21^b^	11.41 ± 0.22^a^	9.34 ± 0.36^b^
URE (IU)	2.52 ± 0.01^c^	2.71 ± 0.01^ab^	2.53 ± 0.02^bc^	2.23 ± 0.03^d^	2.81 ± 0.02^a^	2.81 ± 0.03^a^	2.87 ± 0.02^a^	2.71 ± 0.02^b^	2.66 ± 0.01^b^	2.89 ± 0.03^a^
BG (IU)	2.04 ± 0.02^a^	1.98 ± 0.02^ab^	1.95 ± 0.03^ab^	1.92 ± 0.01^b^	1.99 ± 0.03^ab^	1.98 ± 0.02^ab^	2.04 ± 0.02^a^	1.92 ± 0.01^b^	1.94 ± 0.02^b^	1.94 ± 0.01^b^
BX (IU)	2.06 ± 0.03^a^	2.02 ± 0.02^ab^	1.98 ± 0.03^ab^	1.94 ± 0.007^b^	2.01 ± 0.02^ab^	2.01 ± 0.02^ab^	2.07 ± 0.04^a^	1.95 ± 0.02^b^	1.96 ± 0.01^b^	1.97 ± 0.02^b^
CBH1 (IU)	1.75 ± 0.01^c^	1.89 ± 0.02^a^	1.71 ± 0.01^c^	1.82 ± 0.01^b^	1.72 ± 0.02^c^	1.89 ± 0.01^b^	1.99 ± 0.02^a^	1.75 ± 0.01^c^	1.81 ± 0.03^bc^	1.77 ± 0.01^c^
Pero (IU)	0.27 ± 0.002^b^	0.29 ± 0.003^b^	0.25 ± 0.002^bc^	0.43 ± 0.003^a^	0.23 ± 0.003^c^	0.43 ± 0.004^b^	0.55 ± 0.001^a^	0.34 ± 0.005^b^	0.22 ± 0.004^c^	0.36 ± 0.003^b^
PhOx (IU)	0.21 ± 0.001^a^	0.17 ± 0.003^ab^	0.16 ± 0.004^b^	0.19 ± 0.001^ab^	0.18 ± 0.001^ab^	0.18 ± 0.003^a^	0.17 ± 0.001^a^	0.16 ± 0.003^b^	0.17 ± 0.002^a^	0.19 ± 0.002^a^
Pine soil										
NR (IU)	8.17 ± 0.13^a^	7.40 ± 0.22^b^	7.35 ± 0.11^b^	7.02 ± 0.11^b^	6.99 ± 0.25^b^	8.22 ± 0.09^a^	7.67 ± 0.04^b^	7.25 ± 0.06^bc^	6.98 ± 0.09^c^	6.29 ± 0.03^d^
ALP (IU)	16.04 ± 0.27^b^	16.92 ± 0.25^b^	16.13 ± 0.17^b^	18.01 ± 0.32^a^	15.96 ± 0.35^b^	15.66 ± 0.16^b^	16.68 ± 0.20^ab^	16.74 ± 0.43^ab^	17.70 ± 0.59^a^	15.56 ± 0.37^b^
ACP (IU)	10.62 ± 0.15^b^	11.32 ± 0.12^ab^	10.93 ± 0.17^b^	11.87 ± 0.19^a^	11.16 ± 0.23^ab^	10.48 ± 0.28^b^	10.89 ± 0.22^ab^	11.11 ± 0.24^ab^	11.96 ± 0.16^a^	11.44 ± 0.16^ab^
URE (IU)	2.91 ± 0.03^a^	2.76 ± 0.06^ab^	2.78 ± 0.05^ab^	2.46 ± 0.07^b^	2.67 ± 0.05^b^	2.94 ± 0.07^b^	3.06 ± 0.03^a^	2.89 ± 0.03^b^	3.05 ± 0.06^a^	3.04 ± 0.05^a^
BG (IU)	1.93 ± 0.02^a^	1.94 ± 0.02^a^	1.92 ± 0.01^a^	1.95 ± 0.02^a^	1.94 ± 0.01^a^	2.10 ± 0.04^a^	1.92 ± 0.02^b^	1.94 ± 0.01^b^	1.93 ± 0.04^b^	1.94 ± 0.01^b^
BX (IU)	1.99 ± 0.02^a^	1.98 ± 0.02^a^	1.97 ± 0.01^a^	1.99 ± 0.02^a^	1.98 ± 0.01^a^	2.13 ± 0.04^a^	1.96 ± 0.01^b^	1.98 ± 0.01^b^	1.97 ± 0.04^b^	1.98 ± 0.01^b^
CBH1 (IU)	1.85 ± 0.02^a^	1.69 ± 0.01^b^	1.83 ± 0.01^a^	1.68 ± 0.01^b^	1.81 ± 0.01^a^	1.93 ± 0.02^a^	1.75 ± 0.01^b^	1.88 ± 0.02^a^	1.74 ± 0.02^b^	1.84 ± 0.01^a^
Pero (IU)	0.34 ± 0.004^b^	0.48 ± 0.004^a^	0.43 ± 0.005^a^	0.46 ± 0.003^a^	0.36 ± 0.004^b^	0.59 ± 0.005^a^	0.45 ± 0.005^b^	0.55 ± 0.001^a^	0.47 ± 0.004^b^	0.29 ± 0.002^c^
PhOx (IU)	0.17 ± 0.003^b^	0.22 ± 0.001^a^	0.18 ± 0.001^b^	0.21 ± 0.003^a^	0.17 ± 0.001^b^	0.23 ± 0.001^a^	0.17 ± 0.002^bc^	0.18 ± 0.001^b^	0.17 ± 0.003^bc^	0.16 ± 0.001^c^

*Notes*

Different superscript letters (a, b and c) in a row show significant differences among treatments (*p *<* *0.05).

ACP: acid phosphatase; ALP: alkaline phosphatase; BG: b‐1,4‐glucosidase; BX: b‐1,4‐xylosidase; CBH1: cellobiohydrolase; NR: nitrate reductase; Pero: peroxidase; PhOx: phenol oxidase; URE: urease.

The activity of most soil enzymes responded significantly (Tukey's test, *p *<* *0.05) to soil type, the presence of isopods, the extract treatments, and their interaction. With the exception of ACP and ALP activity, the interaction of isopod presence/absence and treatment was significant for most enzymes (Table 3).

## 4 DISCUSSION

### 4.1 The effects of aqueous extracts on *Q. variabilis* litter decomposition

Previous studies showed that the aqueous extracts prepared from litter reduced soil processes, such as soil C decomposition and N process, suggesting an inhibitory effect of litter secondary compounds (Chomel et al., 2014; Zhang et al., 2014). It was pointed out that secondary compounds may influence soil decomposition through allelopathic effects limiting the growth and activity of decomposers. However, the decomposition study presented here found that, regardless of isopod presence, the addition of high concentrations of aqueous extracts prepared from *P. massoniana* litter increased the decomposition rate of *Q. variabilis* litter when paired with pine (i.e., *P. massoniana*)‐derived soil. This result is opposite to our first hypothesis and previous observation where aqueous litter extracts inhibited decomposition and nutrient cycling (Gonzalezmunoz, Costatenorio, & Espigares, 2012; Zhang et al., 2014). Due to the wide diversity of secondary compounds in nature, it is not surprising that extracts from different species may have different effects on litter decomposition (Kraus et al., 2003). The enhanced decomposition seen here could be explained by the presence of protein or carbohydrate residues in the aqueous extracts; these may act as food for decomposers, enhancing their metabolic activity and enzyme excretion (Aguilera et al., 2015; Kraus et al., 2003). From our results, in the absence of isopods, the addition of high concentrations of aqueous extracts to pine‐derived soil enhanced SIR and both peroxidase and phenol oxidase activity, consequently accelerating the rate of *Q. variabilis* litter decomposition.

### 4.2 The effects of aqueous extracts on isopods activity

Although many soil animals are omnivorous, mounting evidence supports the idea that volatile compounds from leaf litter can directly increase foraging efficiency in insects, perhaps as a result of the identification of a specific food source (Dicke & Baldwin, 2010). It is clear that coniferous leaf litter emits small oxidized volatile organic compounds (Faiola et al., 2014; Ludley, Jickells, Chamberlain, Whitaker, & Robinson, 2009). As the isopods used in this study previously grazed in mixed conifer‐broadleaf forests, they may have developed a preference for the specific volatiles of *P. massoniana* leaf litter, an easy way to find food source. Thus, aqueous extracts would have increased isopod attraction in pine soil, leading to enhanced feeding activity on *Q. variabilis* litter. Aqueous extracts also increased soil pH, which can increase isopod survival (Witt, 1997), again enhancing litter decomposition via a positive effect on isopods.

The high concentration of aqueous extracts also enhanced isopod survival pine soil (compared to controls), again possibly as a result of a greater abundance of proteins or carbohydrates (Aguilera et al., 2015); more live isopods in turn decreased soil microbial biomass, but increased *Q. variabilis* litter decomposition rates. Meehan, Couture, Bennett, and Lindroth (2014) found that isopods could increase microbial biomass via their production of nutrient‐rich frass; typically, isopods are important fungal feeders, regulating microbial biomass and community composition in soil ecosystems (Crowther, Boddy, & Jones, 2011; Mitschunas, Wagner, & Filser, 2006). Here, the soil microbial biomass was reduced by isopod grazing activity, especially in the low aqueous treatment in oak soil and high aqueous treatment in pine soil. This implied that any positive effects of isopods on soil microbes (such as frass provision) were not sufficient to compensate for losses due to predation. In addition, soil enzymes can provide useful information about microbial activity (Chomel et al., 2016; Joanisse, Bradley, Preston, & Munson, 2007). In this study, the activity of most soil enzymes was reduced by the addition of low concentration of aqueous extracts in oak soil. In pine soil, the activity of peroxidase, phenol and carbon‐degrading enzymes was reduced by high concentrations of aqueous extracts; isopod survival was also enhanced by this treatment, suggesting an inhibitive effect of isopod feeding on microbial activity. In other words, the fewer isopods that died, the lower the soil microbial activity and the faster litter decomposed. Hence, with isopods present, secondary compounds may affect *Q. variabilis* litter decomposition rates indirectly via the isopods, rather than via effects on microbes themselves.

### 4.3 The effects of tannins on *Q. variabilis* litter decomposition

In oak‐derived soil, the addition of high concentrations of aqueous extracts or tannins inhibited *Q. variabilis* litter decomposition and soil microbial biomass, regardless of isopod treatment. Previous researches have shown that tannins can limit the growth and activity of decomposers, from microorganisms to soil animals (Asplund, Bokhorst, et al., 2013; Barbehenn & Peter Constabel, 2011; Chomel et al., 2014; Hättenschwiler & Vitousek, 2000). Also, more recalcitrant tannins can decrease the palatability of litter (Asplund, Wardle, & Heil, 2013; Hättenschwiler & Vitousek, 2000; Madritch & Lindroth, 2015), and this may be the case here, with extracts negatively affecting the decomposers of *Q. variabilis* litter. For example, more isopods died with high concentrations of aqueous extracts (contained about 6.4% tannins) or tannins, reducing any feeding effects on litter decomposition (Jia et al., 2015). Tannins can also slow decomposition more directly, via microbial toxicity or by forming recalcitrant complexes with organic N (Kraus et al., 2003; Madritch & Lindroth, 2015), which can inhibit conversion of organic N to inorganic N, especially in broadleaf forest soil (Ushio et al., 2012). Here, nitrogen‐degrading enzymes were inhibited by high concentration aqueous extracts or tannin treatments without isopods, providing support for this explanation. In addition, tannins can reduce soil microbial biomass and reduce the excretion of exo‐enzymes (Kanerva, Kitunen, Kiikkilä, Loponen, & Smolander, 2006; Kraus et al., 2003) that mediate the decomposition of refractory materials such as lignin. For example, without isopods, high tannin treatments significantly decreased the SIR as well as the activity of carbon‐degrading enzymes, such as BX and CBH1, in agreement with previous studies (Kanerva et al., 2006; Kraus et al., 2003; Smolander, Kanerva, Adamczyk, & Kitunen, 2011). Therefore, in oak soil, tannin addition deterred isopods and microorganisms, decelerating *Q. variabilis* litter decomposition. But this was not the case in pine soil, perhaps because pine soil naturally contain some tannins: for example, tannin concentrations as high as 37 mg/g were found in the soils of a Canadian black spruce forest (Lorenz, Preston, Raspe, Morrison, & Feger, 2000). Thus, probably due to long‐term local adaptation of decomposers to tannin toxicity (Chomel et al., 2014), the addition of further tannins (via aqueous or tannin extracts) to pine soil did not inhibit *Q. variabilis* litter decomposition (Ayres, Steltzer, Berg, & Wall, 2009; Chomel et al., 2016). Furthermore, some low molecular weight tannins serve as an active carbon source for soil fauna, which can have a positive effect on soil processes (Fierer, Schimel, Cates, & Zou, 2001) as seen here with higher litter decomposition rates in the high tannins treatment in pine soil with isopods.

## 5 CONCLUSION

With this study, we showed that *Q. variabilis* litter decomposition was enhanced by high concentrations of secondary compounds in pine soil, but decreased in oak soil regardless of isopod activity. Among the secondary compounds found in *P. massoniana* litter, tannins could be a key factor inhibiting soil decomposer activity, thus decreasing *Q. variabilis* litter decomposition in oak soil, but not pine soil where decomposers are well adapted to local resources. Thus, in coniferous forests, the mixture of broadleaf trees could greatly enhance nutrient cycling and ecosystem productivity. In broadleaf forests, mixed coniferous trees would have positive effects on forest nutrient cycling processes.

## CONFLICT OF INTEREST

None declared.

## AUTHOR CONTRIBUTIONS

Hong Lin and Xingjun Tian designed the work. Hong Lin, Yunxia Zhao, Numaimaiti Muyidong, Kai Tian, Zaihua He, and Xiangshi Kong performed the experiments and analyzed the data. Hong Lin and Xingjun Tian wrote the manuscript. Shucun Sun helped revising the manuscript.

## DATA ACCESSIBILITY

The data of figures have been deposited in the FigShare database (https://doi.org/10.6084/m9.figshare.6244373; https://figshare.com/s/b5036c6a0336ef352f4f).
