# Supplementary material for: Secondary compounds of Pinus massoniana alter decomposers' effects on Quercus variabilis litter decomposition
Source: Ecol Evol. 2018 Aug 29;8(18):9439–50. doi: 10.1002/ece3.4433 (PMC6194249; doi:10.1002/ece3.4433)
Supplement: Supplementary file 1 [file ECE3-8-9439-s001.docx]

**Supplemental Material:**

**TABLE S1** Initial chemical properties of *Q. variabilis* and *P. massoniana* litter and the soil characteristics (from one-way ANOVA).

|  | *Q. variabilis* | *P. massoniana* |
| --- | --- | --- |
| Litter |  |  |
| Total C (%) | 46.30^a^ | 31.97^b^ |
| Total N (%) | 0.75^a^ | 0.37^b^ |
| Lignin (%) | 30.52^b^ | 41.20^a^ |
| C/N | 61.73^b^ | 86.41^a^ |
| Lignin/N | 40.69^b^ | 111.35^a^ |
| Tannins (%) | 1.02^b^ | 6.4^a^ |
| Soils | oak soil | pine soil |
| pH | 4.40^b^ | 4.62^a^ |
| Total C (%) | 4.17^b^ | 4.69^a^ |
| Total N (%) | 0.27^a^ | 0.28^a^ |
| C/N | 15.4^b^ | 16.8^a^ |

Data with different superscript letters in row are significantly different (*p*<0.05, *n*=4).

**TABLE S2** The pH of oak soil and pine soil with and without isopod under different treatments after six months incubation.

| pH | oak soil | pine soil |
| --- | --- | --- |
| Without isopods |  |  |
| Control | 4.22^b^ | 4.30^b^ |
| High aqueous | 4.23^b^ | 4.47^a^ |
| Low aqueous | 4.31^a^ | 4.31^b^ |
| High tannins | 4.05^c^ | 4.19^c^ |
| Low tannins | 4.24^b^ | 4.32^b^ |
| With isopods |  |  |
| Control | 4.27^b^ | 4.30^b^ |
| High aqueous | 4.31^b^ | 4.68^a^ |
| Low aqueous | 4.46^a^ | 4.38^b^ |
| High tannins | 4.09^c^ | 4.15^c^ |
| Low tannins | 4.30^b^ | 4.36^b^ |

Data with different letters in a vertical row indicates a significant difference (*p* < 0.05) from ANOVA with repeated measurements.
